# Surveillance of Human Guinea Worm in Chad, 2010–2018

**DOI:** 10.4269/ajtmh.20-1525

**Published:** 2021-05-24

**Authors:** Sarah Anne J. Guagliardo, Ernesto Ruiz-Tiben, Donald R. Hopkins, Adam J. Weiss, Philippe Tchindebet Ouakou, Hubert Zirimwabagabo, Karmen Unterwegner, Dillon Tindall, Vitaliano A. Cama, Henry Bishop, Sarah G. H. Sapp, Sharon L. Roy

**Affiliations:** 1 Parasitic Diseases Branch, Division of Parasitic Diseases and Malaria, Centers for Disease Control and Prevention, Atlanta, Georgia;; 2 Guinea Worm Eradication Program, The Carter Center, Atlanta, Georgia;; 3 Guinea Worm Eradication Program, Ministry of Public Health, N’Djamena, Chad

## Abstract

The total number of Guinea worm cases has been reduced by 99.9% since the mid-1980s when the eradication campaign began. Today, the greatest number of cases is reported from Chad. In this report, we use surveillance data collected by the Chad Guinea Worm Eradication Program to describe trends in human epidemiology. In total, 114 human cases were reported during the years 2010–2018, with highest rates of containment (i.e., water contamination prevented) in the years 2013, 2014, 2016, and 2017 (*P* < 0.0001). Approximately half of case-patients were female, and 65.8% of case-patients were aged 30 years or younger (mean: 26.4 years). About 34.2% of case-patients were farmers. Cases were distributed across many ethnicities, with a plurality of individuals being of the Sara Kaba ethnicity (21.3%). Most cases occurred between the end of June and the end of August and were clustered in the Chari Baguirmi (35.9%) and Moyen Chari regions (30.1%). Cases in the northern Chari River area peaked in April and in August, with no clear temporal pattern in the southern Chari River area. History of travel within Chad was reported in 7.0% of cases, and male case-patients (12.5%) were more likely than female case-patients (1.7%) to have reported a history of travel (*P* = 0.03). Our findings confirm that human Guinea worm is geographically disperse and rare. Although the proportion of case-patients with travel history is relatively small, this finding highlights the challenge of surveillance in mobile populations in the final stages of the global eradication campaign.

## INTRODUCTION

The global Guinea worm eradication program represents one of the great public health success stories of our time. Since the program’s inception in 1980, the number of annual human cases has reduced by 99.9%, from an estimated 3.5 million cases in 21 countries^1^ to only 54 cases in three countries at the end of 2019.^2,3^

Causing significant disability and pain, Guinea worm infection occurs through the ingestion of freshwater copepods (small water crustaceans) infected with stage 3 *Dracunculus medinensis* larvae. After approximately 10–14 months, a blister forms (usually on a lower limb) where the pregnant adult female Guinea worm ruptures the skin.^4^ Upon detection of a possible Guinea worm case, patients are transported to health centers where they receive care. The worm is extracted via “controlled immersion,” in which the wound is submerged in a water-filled container to induce worm emergence without the risk of contaminating public water sources.^5^

Guinea worm is a disease of poverty, primarily impacting people in remote areas who do not have access to safe water for drinking and are forced to drink from stagnant water sources such as ponds, pools, and unprotected open wells that may contain infected copepods.^5^ The seasonality of disease incidence varies geographically owing to the timing of precipitation patterns and abundance of stagnant water sources.^5^ For example, in the Sahel (Mali, Niger, Chad), cases historically peaked in the months May through October when stagnant water sources are most abundant. Young adults (15–45 years of age) are most likely to be infected with *D. medinensis*, although persons of all ages can develop infection.^5^ Guinea worm cases have historically been equally distributed among men and women, though some exceptions have been noted.^5^ Occupation is an important risk factor; farmers and persons who fetch drinking water are commonly infected.^5^ In some countries (i.e., Mali, Niger, and Burkina Faso), certain ethnic groups are at higher risk of infection because of seasonal migration across long distances and, in some cases, the seasonal search for water or pasture for cattle.^5^

Conventional thought about Guinea worm transmission ecology was challenged in 2012 when canine cases were first reported in Chad in significant numbers,^6^ spurring new research on transmission dynamics. Today, Chad is the epicenter of *D. medinensis* transmission, reporting 88% of human cases (*N* = 48) and virtually all canine cases (99%, *N* = 1,927) worldwide in 2019.^2,3,7^ Recent case-control studies conducted in Chad have shown that water sources associated with increased risk for Guinea worm include lagoons, ponds, and untreated water from hand-dug wells.^8,9^ No associations have been observed to date between human Guinea worm and consumption of fish and frogs,^9^ which has been demonstrated in dogs.^10^ This is could be because humans are less likely than dogs to consume uncooked small fish/fingerlings and frogs, which can serve as transport or paratenic hosts.^11,12^

Over five years have passed since Eberhard et al.^6^ first described the unusual epidemiological patterns in Chad, noting that human cases are sporadic and rare in comparison with canine cases, with no apparent association with common water sources and no clustering by village. Since that time, the number of canine cases has generally increased steadily as surveillance expanded,^2,3,13^ whereas human cases have remained generally constant and at low numbers.^2,3^ Here, we use additional surveillance data to determine whether those epidemiologic patterns persist and to characterize in greater depth human Guinea worm in Chad, with the broader goal of furthering our understanding *D. medinensis* transmission and epidemiology.

## METHODS

### Ethical considerations.

Data collected for this analysis is part of routine public health surveillance conducted by the Chadian Ministry of Public Health. Analysis of Guinea worm surveillance data was deemed not to be research as defined in 45 CFR 46.102(l) by the delegated authority at the CDC Center for Global Health (project ID: 0900f3eb819c65ad), and institutional review board review was not required.

### Surveillance system.

We analyzed human data collected during 2010–2018 through the Chad Guinea Worm Eradication Program (CGWEP) surveillance system, described in detail elsewhere.^13^ Although data from 2019 were available, we excluded that year from this analysis because cases affected by a water-borne outbreak^3,14^ would skew our exploration of general trends.

Briefly, CGWEP surveillance is organized in three levels of intensity.^13^ A cash reward program is in place at all surveillance levels to incentivize community members to report rumors of suspect Guinea worm cases in humans and animals.^6^ Levels 1 and 2 are active systems, in which village-level volunteers conduct daily household searches for possible cases of disease in both humans and animals. Level 1 surveillance areas are in locations where disease has occurred, and Level 2 areas are in geographic proximity to Level 1 surveillance areas. Level 1 has more frequent and intense supervision of volunteers by field teams. In 2019, there were about 2,300 villages under active surveillance in Chad. Level 3 surveillance is a passive system, in which rumors or cases are detected through the Integrated Disease Surveillance and Response System of the existing Ministry of Public Health infrastructure. Level 3 surveillance occurs in all areas of Chad not under Level 1 or 2 surveillance.

For each human case, CGWEP collects information on demographics (e.g., age, sex, ethnicity), occupation, location, migration history, case management information (including containment information; see below), and the number of emergent worms, among other variables. A complete list of variables that are available for analysis by year is shown in Supplemental Table 1.

### Containment criteria.

A Guinea worm is considered to be “contained” when 1) the case is detected and treated within 24 hours of emergence; 2) the patient did not enter a water source with an emerging worm; 3) the lesion was properly cleaned and bandaged until the worm removal and health education has been provided to the patient; 4) the containment process, including verification of dracunculiasis, is visually validated by a CGWEP supervisor within seven days of worm emergence; and 5) the chemical temephos (Abate^®^, Raleigh, NC) is used to treat any contaminated water sources.^3^ If a case-patient has multiple worms, containment is only achieved when all worms from that patient have been extracted and only if all the worms emerging during that calendar year have been contained.^3^

### Confirmatory testing.

A case of Guinea worm disease is defined as “a person exhibiting a skin lesion with emergence of a Guinea worm, ideally with laboratory confirmation.”^15^ Upon extraction, suspected Guinea worms are sent to the CDC Parasitic Diseases laboratory in Atlanta, Georgia for confirmatory testing. Worm specimens undergo morphological examination, and when microscopy results are equivocal (usually due to poor specimen condition), polymerase chain reaction tests and DNA sequencing are conducted for species determination.^16^ Test results are then reported to the national GWEP via The Carter Center headquarters and to WHO headquarters and its regional offices, who in turn relay results to the in-country point-of-contact to disseminate to the field level. Upon positive laboratory confirmation, a village ceremony is held to award the patient with a cash reward to encourage other residents to identify and report future cases.

### Data analysis.

Data management and analyses were conducted in SAS 9.3 (SAS, Cary, NC), and graphs were developed in the R base package.^17^ We conducted descriptive analyses of 1) Guinea worms extracted from humans, 2) human patients with Guinea worm, and 3) the spatial and temporal distribution of Guinea worm cases.

We tallied the total number of confirmed Guinea worms extracted from humans and calculated the proportion of contained Guinea worms by year and the proportion of worms emerging from different locations on the patient’s body. We assessed surveillance system effectiveness by calculating the proportion of cases contained by year, surveillance level in the village at the time of detection (active versus passive), and area within Chad (Fisher’s exact test, *P* < 0.05). Descriptive statistics were also calculated for other variables collected by the surveillance system, including demographic information (age, sex, ethnicity) and whether the patient may have contaminated a drinking water source.

We calculated the frequency of different occupations among the case-patients. This information was originally collected in an open-ended manner, but we divided occupations into different categories for ease of interpretation. When multiple occupations were listed (e.g., farmer and fisherman), both occupations were combined into a single category. We also assessed whether the patient had a history of travel (being physically away from the site of detection for > 1 night) during the period of infection (i.e., the 10–14 months prior to worm emergence). Ethnicities were categorized in congruence with the most recent Demographic Health Survey conducted in Chad in 2014–2015.^18^ To identify possible instances of recurrent infection in Guinea worm case-patients, we conducted a fuzzy matching algorithm using the R package *stringdist*.^19^ The Jaro-Winkler method was used to generate a distance matrix of all unique names (character strings) and classify names into alike clusters. Clusters with more than one name were then assessed to determine whether the geographic location (village) matched all names within the cluster. An individual person was considered to have experienced a recurrent Guinea worm infection only when two instances of similar names were found in the data and the village name was the same for each record.

The temporal distribution of Guinea worm-infected patients was assessed by producing an epidemic curve of cases by month and year. Following the approaches of previous studies,^20^ subregional incidence by month was investigated by dividing villages into northern and southern Chari River areas at 9.56° latitude to reflect parasite population differentiation.^21^ We also mapped villages where cases were identified in Quantum GIS^22^ and calculated the proportions of cases from different regions within Chad. We created pie chart maps at the village level to explore the geographic distributions of occupations and ethnicities among infected persons. For occupations, we focused on the categories of fishermen, farmers, or individuals engaged in both activities because of possible exposures to fish or frogs, which can serve as paratenic or transport hosts.^11,12^ Finally, we tallied the number of cases and villages occurring < 50 km from the borders with Cameroon and the Central African Republic.

## RESULTS

### Characteristics of Guinea worms.

**Figure 1.**
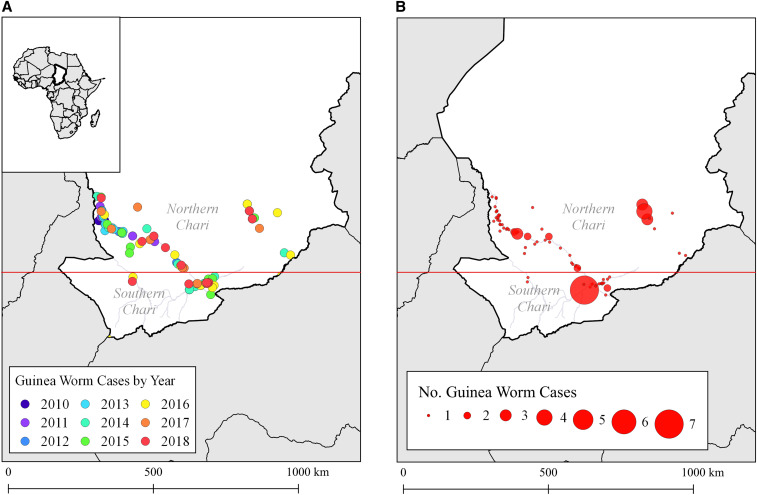
(**A**) Number of Guinea worm cases by year and (B) number of Guinea worm cases by village (**B**), Chad, 2010–2018. Most human cases detected during the period of interest are distributed along the Chari River, which spans from Lake Chad to the southeastern border. The dark red line distinguishes the northern Chari area and the southern Chari area. This figure appears in color at www.ajtmh.org.

In total, 174 laboratory-confirmed *D. medinensis* worms were detected in 114 persons in southern Chad during the years 2010–2018 (Figure 1). The number of worms detected per year ranged from 11 to 32. Similar to worm distribution in dogs,^23^ the number of worms per case-patient was highly aggregated and ranged from 1 to 9 (mean: 1.5; median: 1.0). Most patients (72.8%) presented with a single worm, and 27.2% had multiple worms. Approximately 90% of worms emerged on a patient’s legs; 7% emerged on the arms; and 3% and 1% of worms emerged on the trunk and head/neck, respectively.

**Figure 2.**
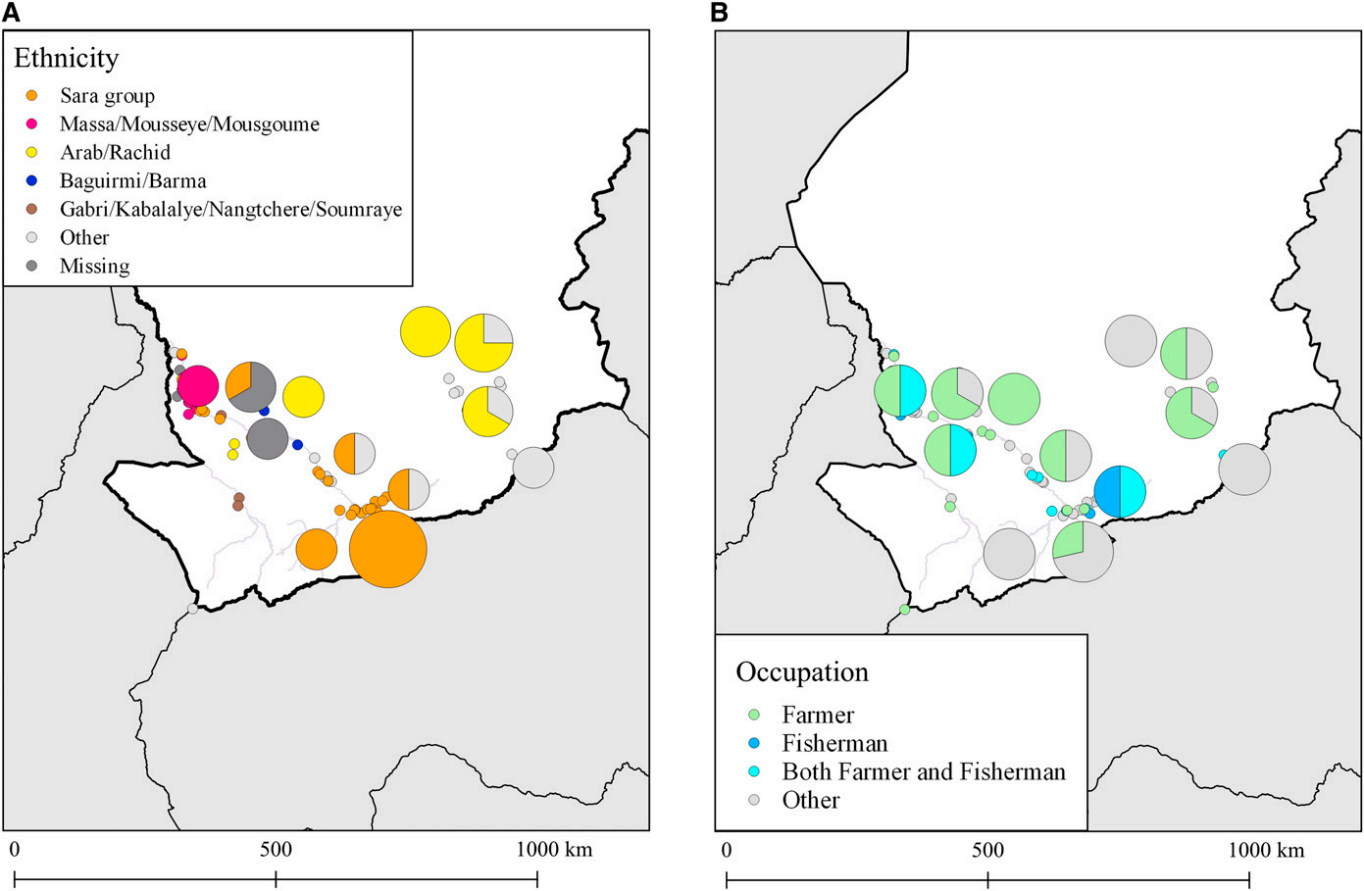
(**A**) Ethnicities and (**B**) occupations of Guinea worm case-patients, Chad, 2010–2018. The Sara ethnic group was distributed along the Chari River and concentrated in the south. Expectedly, fishermen were located near rivers. This figure appears in color at www.ajtmh.org.

The probability of containment of Guinea worm cases varied significantly by year (*P* < 0.0001; Table 1), with highest rates of containment in the years 2013, 2014, 2016, and 2017. The proportion of worms that were successfully contained did not vary significantly by year (χ^2^ = 6.9; *P* = 0.33). Reasons for unsuccessful containment included possible water contamination (29.9%), late reporting of a lesion (> 24 hours after emergence) (28.0%), late or irregular bandaging of a lesion (11.2%), and the worm not being confirmed by a supervisor within the required timeframe (8.4%) (Table 2).

**Table 1:** Proportion of cases contained by year, 2010–2018

Year	*N*	n (%)	*P* value*
2010	10	0 (0)	
2011	10	0 (0)	
2012	10	4 (40)	
2013	14	8 (57.1)	
2014	13	8 (61.5)	
2015	9	0 (0)	
2016	16	9 (56.3)	
2017	15	9 (60)	
2018	17	7 (41.2)	
All years	114	45 (39.5)	**< 0.0001****†**

* Fisher’s exact test.

† Bold indicates statistical significance (*P* < 0.05).

**Table 2:** Reasons for unsuccessful worm containment

Reason for unsuccessful containment	Total observations	*N* (%)
Case possibly contaminated water	134*	40 (29.9)
Late reporting of lesion†	107‡	30 (28.0)
Late or irregular bandaging of a lesion	107‡	12 (11.2)
Worm not confirmed by a supervisor§	107‡	9 (8.4)

* Variable collected 2010–2018.

† More than 24 hours after emergence.

‡ Variable collected 2014–2018.

§ Worm was not confirmed by a supervisor within the required timeframe. All worms were later laboratory confirmed to be *Dracunculus medinensis*.

### Characteristics of Guinea worm case-patients.

There were slightly more female (*N* = 58, 50.9%) Guinea worm case-patients than male case-patients (*N* = 56, 49.1%). The average age of infected persons was 26.0 years (median: 20 years). Patient age remained stable over time (Kruskal-Wallis χ^2^ = 10.4; *P* = 0.24), and 65.8% of cases occurred in individuals aged 30 years or younger. Approximately 63.4% of cases detected in villages under active surveillance were contained, in contrast with 36.6% of contained cases detected in villages under passive surveillance (*P* = 0.0084) (Supplemental Table 2). Containment rates did not vary significantly by age, sex, or region within Chad.

Patients of the Sara ethnic group constituted a plurality (40.4%) of the 94 individuals infected with Guinea worm disease during 2012–2018, the years during which information on ethnicity was collected (Table 3). Other ethnic groups included the Massa/Mousseye/Mousgoume (*N* = 15, 16.0%) and the Arab/Rachid (*N* = 13, 13.8%). About 13% of Guinea worm case-patients reported occupations related to fishing, and about one-third (*N* = 39, 34.2%) were engaged in farming-related occupations (Table 4).

**Table 3:** Characteristics of case-patients, 2012–2018

Characteristic	*N* (%)
Sex	
Female	58 (50.9)
Male	56 (49.1)
Age (years)	
≤ 15	46 (40.4)
16–30	29 (25.4)
31–45	21 (18.4)
45–60	12 (10.5)
> 60	6 (5.3)
Ethnicity*	
Sara group†	38 (40.4)
Massa/Mousseye/Mousgoume	15 (16.0)
Arab/Rachid	13 (13.8)
Baguirmi/Barma	4 (4.3)
Gabri/Kabalalye/Nangtchere/Soumraye	4 (4.3)
Other‡	20 (21.3)
Missing	20
Travel history	
Yes	8 (7.0)
No	106 (93.0)

* Ethnic categories adopted from the most recent Demographic Health Survey (2014).

† The Sara group encompasses many sub-ethnicities, including Sara Kaba, Sara Madjigay, Sara Mousgoum, Sara Kaba Rodjo, Goulaye, Mbaye, Mberi, Mouroum, Ngambaye, and Ngor.

‡ Other ethnicities reported included Rounga (*N* = 3), Kibet (*N* = 2), Loua (*N* = 2), Boa (*N* = 1), Briguite (Abdeya) (*N* = 1), Foulata (*N* = 1), Laka (*N* = 1), Mboulou (*N* = 1), Ndam (*N* = 1), Rouga (*N* = 1), Boulala (*N* = 2), Dadjo (*N* = 1), Hemat (*N* = 1), Mongo (*N* = 2).

**Table 4:** Occupations of Patients Infected with *Dracunculus medinensis* in Chad, 2010–2018

Occupation*	*N* (%)
Farmer only	25 (21.9)
Housewife only	24 (21.1)
Child	21 (18.4)
Student only	17 (14.9)
Farmer + fisherman†	9 (7.9)
Fisherman only	6 (5.3)
Farmer + another profession‡	5 (4.4)
Merchant only	3 (3.5)
Other occupation§	4 (2.6)
Total patients	114 (100)

* Questioning related to occupations is collected in an open-ended manner, and the categories below directly correspond with answers provided by Guinea worm case-patients. If a Guinea worm case-patient indicated that they were a farmer, then that person was counted in the “Farmer only” category. If a case-patient listed “Farmer and fisherman” as their occupation, then that individual was counted in the “Farmer + fisherman” category.

† One person included in the farmer + fisherman group also listed herding as an occupation.

‡ Includes farmer/trader (1), farmer/housewife (1), farmer/student (3).

§ Other occupations include a nomadic herder (1), mason (1), butcher (1), and potter (1).

A history of travel within Chad during the period of infection (the 10–14 months prior to worm emergence) was reported in 8 of 114 cases (7.0%) (Table 5). Male case-patients were significantly more likely than female case-patients to have a history of travel during the period of infection (12.5% versus 1.7%, respectively; χ^2^ = 5.1; *P* = 0.02). Three case-patients with a history of travel had uncontained infections. One case-patient detected in 2011 was a nomadic herder and reported visiting four different villages during the period of infection in addition to five other villages outside the period of infection. This individual’s worm extraction was conducted in a location different from their listed village of residence.

**Table 5:** Guinea worm case-patients with a history of travel

Case-patient	Year	Age (years)	Sex	Ethnicity	Occupation	No. of worms	Containment status	Village of detection
1	2010	4	Female	Unknown	Child	1	Unknown	Moulkou
2	2011	38	Male	Unknown	Nomadic herder	1	Unknown	Moto
3	2012	24	Male	Gabri	Farmer	2	Unknown	Bouram Foulbé
4	2012	57	Male	Boulala	Fisherman	1	Unknown	Hilélé
5	2014	20	Male	Rouga	Butcher	1	Not contained	Am-Bissirigne
6	2014	11	Male	Massa	Child/ student	1	Contained	Bongor
7	2014	40	Male	Mongo	Mason	2	Not contained	Kalam Kalam
8	2015	18	Male	Arabe	Farmer	1	Not contained	Ferick Tchaguine

We found no evidence of recurrent Guinea worm infection over multiple years in any individual patients using the character string matching algorithm used.

### Temporal and spatial distribution of Guinea worm cases.

**Figure 3.**
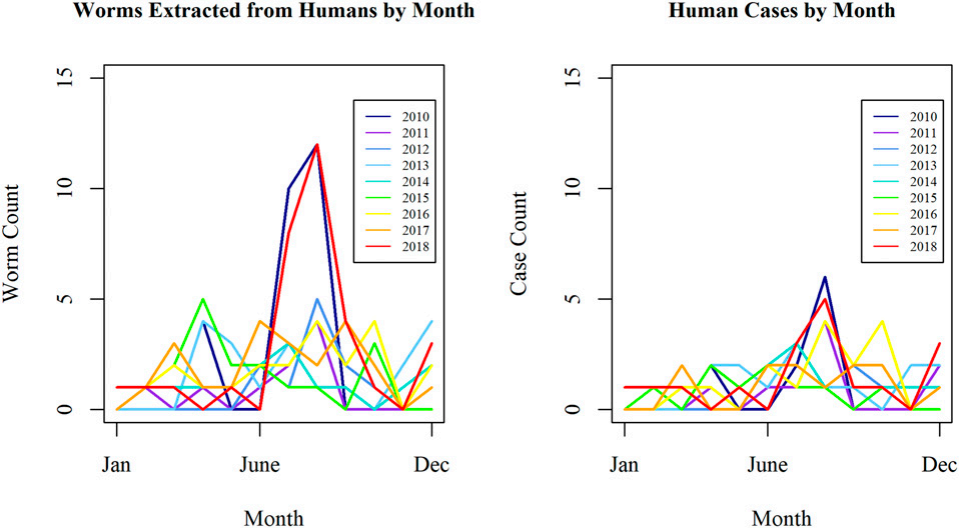
Numbers of Guinea worms and human cases detected by month and year, Chad, 2010–2018. Human Guinea worm cases (and worms) occurred during all months throughout 2010–2018, with most cases occurring between the end of June and the end of August. This figure appears in color at www.ajtmh.org.

The greatest numbers of both Guinea worms and infected people were detected between the end of June and the end of August (Figure 3). The northern Chari River area saw a more marked seasonal pattern relative to the southern Chari River area, with peak numbers of worms and cases occurring in the months of April and August (Supplemental Figure 1). Cases occurred in the Regions of Chari Baguirmi (*N* = 38, 35.9%), Moyen Chari (*N* = 33, 31.1%), Salamat (*N* = 19, 17.9%), and Mayo Kebbi Est (*N* = 10, 9.4%), with a few also occurring in Tandjile (*N* = 3, 2.8%), Mandoul (*N* = 2, 1.9%), and Logone Oriental (*N* = 1, 0.9%) (Supplemental Figure 2).

Cases were distributed across 89 villages, and the maximum total number of cases per village was seven (Maimou in 2013 [*N* = 5] and 2014 [*N* = 2]). Approximately 55% of villages reporting human cases had access to at least one potable water source (e.g., at least one borehole well with a functional pump).

Clear geographic patterns were observed among case-patients of different ethnicities. People belonging to the Sara group ethnicity were largely found along the Chari River, and people in the Massa/Mousseye/Mousgoume group were located in the northern Chari River area. The “middle” Chari River area in between the north and south was characterized by more diversity, where the Arab/Rachid, Baguirmi/Barma, and Gabri/Kabalalye/Nangtchere/Soumraye people were located (Figure 2A). Among the case-patients, farmers were found throughout southern Chad, whereas fishermen and people who were both farmers and fishermen were located close to the Chari River and its tributaries (Figure 2B).

Thirty-three cases occurred in 31 villages located within 50 km of Chad’s borders with Cameroon and the Central African Republic. Among these villages, five were within 20 km of Chad’s borders.

## DISCUSSION

Despite the eradication program’s remarkable global success, challenges in the final stages of the campaign have been widely acknowledged, including the eradication of disease in animals,^6,13,24^ the recent identification of paratenic and transport hosts in the transmission cycle,^11,12^ and insecurity.^24,25^ In this report, we call attention to the additional challenge of surveillance in mobile populations, in terms of movement of individuals and groups within Chad, and transboundary movement between Chad and bordering nations.

Our results showed that approximately 7% of Guinea worm case-patients reported a history of travel (being physically away from the site of detection for more than one night) during the period of infection. Among these persons, 37.5% had uncontained infections, suggesting that mobile people could contaminate water sources along travel routes. Although the overall proportion of case-patients with travel history is small, the surveillance system could improve notation of travel history. Consultation with CGWEP field staff revealed that one case-patient infected in 2011 was subsequently reinfected in 2013 in a different village. This was not detected by our string-matching algorithm because it relied on name-matching by village, which assumes that people remain in the same location from year to year. Adding detailed questions to the case surveillance form about travel history across years, places that they have lived in the past, presumed source of infection,^26^ and about reinfection would facilitate detection of persons residing in one location that later move to another location.

Nomadic pastoralists in particular may warrant heightened surveillance because in Chad it is estimated that these peoples comprise anywhere from 400,000^27^ to 2 million individuals.^28^ Our findings showed that one case-patient was a nomadic herder by trade, visiting a total of nine locations in the 14 months prior to the emergence of the worm. Two other case-patients were detected in villages that contained the terms “Ferrick” or “Foulbé,” which imply association with nomadic camps or the Foulbé/Fulani nomadic pastoralist ethnicity, respectively. We also note that 30 of 2,752 villages under active surveillance during 2010–2018 contained the terms “Ferrick” or “Foulbé”; among these villages, two reported dog infections. The CGWEP is structured around detecting infections in permanent settlements, but from the standpoint of disease eradication, nomadic groups may require special attention because these persons have the potential to carry infection across large geographic areas. Although challenging,^29^ surveillance of Guinea worm in nomadic groups, combined with mapping nomadic routes and tracing transmission through *D. medinensis* population genetics,^21^ could be helpful. In 2020, the CGWEP began recruiting volunteers from within mobile populations in Moyen Chari Region to implement active surveillance in those groups while also ensuring community acceptance and engagement. Plans are currently underway to expand active surveillance to mobile communities in other regions.

The occurrence of 33 human cases near Chad’s borders is of concern and raises questions about transboundary spread between Chad, Cameroon, and the Central African Republic. The World Health Organization certified the Central African Republic to be free of Guinea worm in 2006, but insecurity in that country remains a challenge to any public health surveillance.^24^ This is likely particularly true for the surveillance of dogs, in which Guinea worm is more difficult to detect. Cameroon was also certified to be free of Guinea worm in 2007, but, regretfully, four canine cases and one feline case were reported in 2020, in addition to a human case that was likely infected in Chad.^30,31^ In response, active surveillance has been initiated in communities on both sides of the Chad-Cameroon border, along with application of Abate to water sources, tethering of dogs, purchasing and destroying fish guts, and health messaging campaigns.^30^

*Dracunculus medinensis* infection occurs in a range of hosts in Chad, including humans and domestic dogs and cats,^6^ and evidence from population genetics and genomics shows that worms emerging from these hosts are indeed the same population.^21,31^ Some scholars have called for a One Health approach to eradication, including the need to rigorously surveil all hosts, characterize spatial and temporal overlap of infection in all hosts, and determine risk factors for transmission from animals to humans.^31,32^ In this analysis we explore the human epidemiology of the Guinea worm in detail without considering the full context of canine and feline hosts, largely because data about worms extracted from different hosts were collected independently, devoid of linking identifiers between datasets. Therefore, at present there is no way to accurately link human case-patients with owners of infected dogs or owners of infected dog with owners of infected cats. This is a future priority, and several improvements to the information system are currently underway, such as transition from paper-based to electronic data capture, individual tracking of dogs via ID cards, and implementation of household-level identifiers. Although we can furnish some contrasts between human and canine hosts (e.g., ethnic diversity and occupational diversity among human cases is greater than for owners of infected dogs^13^) synchronizing data about worms extracted from humans, dogs, and cats may yield additional insights into the *D. medinesis* transmission system.

Importantly, this analysis does not reveal insights about risk factors for human Guinea worm because the CGWEP only collects information on confirmed cases. Surveillance data, however, could be improved by collecting more detailed information about travel and migration history, in addition to information about exposures including consumption of contaminated water, dietary habits, and epidemiological and social links between human cases and infected animals (e.g., asking Guinea worm patients about dog and cat ownership and history of infection in their animals). Previous infection in humans is a strong correlate of subsequent infection.^33^ It would therefore also be useful to gather information on infection history for individuals and within families rather than using fuzzy matching algorithms to identify similar names that appear multiple times within the dataset. Information about water sources could also be important because case-control studies in Chad showed that unprotected wells and secondary water sources (i.e., water sources located outside the village or other sources used at home excluding the main water supply) were a significant risk factor for human Guinea worm.^8,9^ Lastly, the uneven coverage of surveillance over space and time could impact our ability to accurately assess trends. In this analysis, we found that only about 30% of human Guinea worm cases were detected in villages under active surveillance at the time of case presentation. A 2019 CGWEP surveillance system evaluation found that the frequency of volunteer visits to households was varied despite program guidance that requires that volunteer visits are to be conducted on a daily basis.^34^ Improved supervision and volunteer training and consistent application of program standards throughout different times of year have been identified as future priorities for the CGWEP.^34^

Until full control of the zoonotic reservoir(s) is achieved in Chad, renewed transmission to humans will likely remain a risk. This is further complicated by the additional difficulty of detecting and containing infections in mobile groups of people, and by extension their dogs, underscoring the need for a multipronged approach to surveillance and control of Guinea worm.

## Supplemental information, tables, and figure

Supplemental materials

## References

[1] Watts SJ, 1987. Dracunculiasis in Africa in 1986: its geographic extent, incidence, and at-risk population. Am J Trop Med Hyg 37: 119–125. 2955710 10.4269/ajtmh.1987.37.119

[2] Hopkins DR Ruiz-Tiben E Weiss AJ Roy SL Zingeser J Guagliardo SAJ, 2018. Progress toward global eradication of dracunculiasis—January 2017–June 2018. MMWR 67: 1265–1270. 30439874 10.15585/mmwr.mm6745a3 PMC6290806

[3] Hopkins DR Weiss AJ Roy SL Zingeser J Guagliardo SAJ, 2019. Progress toward global eradication of dracunculiasis—January 2018–June 2019. MMWR Rep 68: 979–984. 10.15585/mmwr.mm6843a5 PMC6822808 31671082

[4] Muller R, 1971. Dracunculus and dracunculiasis. Adv Parasitol 9: 73–151. 4252302 10.1016/s0065-308x(08)60160-8

[5] Ruiz-Tiben E Hopkins DR, 2006. Dracunculiasis (Guinea worm disease) eradication. Adv Parasitol 61: 275–309. 16735167 10.1016/S0065-308X(05)61007-X

[6] Eberhard ML 2014. The peculiar epidemiology of dracunculiasis in Chad. Am J Trop Med Hyg 90: 61–70. 24277785 10.4269/ajtmh.13-0554 PMC3886430

[7] World Health Organization Collaborating Center for Dracunculiasis Eradication, 2020. *Guinea Worm Wrap-up #265*. Available at: https://www.cartercenter.org/resources/pdfs/news/health_ publications/guinea_worm/wrap-up/265.pdf. Accessed November 19, 2020.

[8] Sreenivasan N Weiss A Djiatsa JP Toe F Djimadoumaji N Ayers T Eberhard M Ruiz-Tiben E Roy SL, 2017. Recurrence of Guinea worm disease in Chad after a 10-year absence: risk factors for human cases identified in 2010–2011. Am J Trop Med Hyg 97: 575–582. 28722616 10.4269/ajtmh.16-1026 PMC5544091

[9] Liu EW 2020. Investigation of Dracunculiasis transmission among humans, Chad, 2013–2017. Am J Trop Med Hyg 104: 724–730. 10.4269/ajtmh.20-0584 PMC7866328 33289475

[10] McDonald RA Wilson-Aggarwal JK Swan GJF Moundai T Sankara D Biswas G Zingeser J, 2020. Ecology of domestic dogs *Canis familiaris* as an emerging reservoir of Guinea worm *Dracunculus medinensis* infection. PLoS Negl Trop Dis 14: e0008170. 32310976 10.1371/journal.pntd.0008170 PMC7170223

[11] Cleveland CA Eberhard ML Thompson AT Smith SJ Zirimwabagabo H Bringolf R Yabsley MJ, 2017. Possible role of fish as transport hosts for *Dracunculus* spp. larvae. Emerg Infect Dis 23: 1590–1592. 28820381 10.3201/eid2309.161931 PMC5572877

[12] Eberhard ML Yabsley MJ Zirimwabagabo H Bishop H Cleveland CA Maerz JC Bringolf R Ruiz-Tiben E, 2016. Possible role of fish and frogs as paratenic hosts of *Dracunculus medinensis*, Chad. Emerg Infect Dis 22: 1428–1430. 27434418 10.3201/eid2208.160043 PMC4982183

[13] Guagliardo SAJ Roy SL Ruiz-Tiben E Zirimwabagabo H Romero M Chop E Ouakou PT Hopkins DR Weiss AJ, 2020. Guinea worm in domestic dogs in Chad: a description and analysis of surveillance data. PLoS Negl Trop Dis 14: e0008207. 32463811 10.1371/journal.pntd.0008207 PMC7255611

[14] World Health Organization Collaborating Center for Dracunculiasis Eradication, 2019. *Guinea Worm Wrap-up #263*. Available at: https://www.cartercenter.org/resources/pdfs/news/health.publications/guinea_worm/wrap-up/263.pdf. Accessed November 19, 2020.

[15] World Health Organization, 2020. *Dracunculiasis Eradication*. Available at: https://www.who.int/dracunculiasis/surveillancecontrol/control_and_surveillance_surveillance/en/. Accessed November 19, 2020.

[16] Bimi L Freeman AR Eberhard ML Ruiz-Tiben E Pieniazek NJ, 2005. Differentiating *Dracunculus medinensis* from *D. insignis*, by the sequence analysis of the 18S rRNA gene. Ann Trop Med Parasitol 99: 511–517. 16004710 10.1179/136485905X51355

[17] R Core Development Team, 2019. R: A Language and Environment for Statistical Computing. Vienna, Austria: R Foundation for Statistical Computing.

[18] Institut National de la Statistique, des E’ tudes E’ conomiques et De’mographiques (INSEED), Ministe’ re de la Sante’ Publique (MSP), 2016. *Enqueˆ te De’mographique et de Sante’ et a’ Indicateurs Multiples, 2014–2015*. Fairfax, VA: ICF International.

[19] van der Loo MK, 2014. The stringdist package for approximate string matching. *R J 6*:111–122.

[20] Richards RL Cleveland CA Hall RJ Ouakou PT Park AW Ruiz-Tiben E Weiss A Yabsley MJ Ezenwa VO, 2020. Identifying correlates of Guinea worm (*Dracunculus medinensis*) infectionin domestic dog populations. PLoS Negl Trop Dis 14: e0008620. 32925916 10.1371/journal.pntd.0008620 PMC7515199

[21] Thiele EA Eberhard ML Cotton JA Durrant C Berg J Hamm K Ruiz-Tiben E, 2018. Population genetic analysis of Chadian Guinea worms reveals that human and non-human hosts sharecommon parasite populations. PLoS Negl Trop Dis 12: e0006747. 30286084 10.1371/journal.pntd.0006747 PMC6191157

[22] QGIS Development Team, 2020. QGIS Geographic Information System. OpenSource Geospatial Foundation Project. Available at: http://www.qgis.org. Accessed November 25, 2020.

[23] Guagliardo SAJ Wiegand R Roy SL Cleveland CA Zirimwabagabo H Chop E Ouakou PT Ruiz-Tiben E Hopkins DR Weiss AJ, 2021. Correlates of variation in Guinea worm burden among infected domestic dogs. Am J Trop Med Hyg 104: 1418–1424. 10.4269/ajtmh.19-0924 PMC8045642 33617473

[24] Molyneaux DH Eberhard ML Cleaveland S Addey R Guiguemde RT Kumar A Magnussen P Bremen JG, 2020. Certifying Guinea worm eradication: current challenges. Lancet 396: 1857–1860. 33278938 10.1016/S0140-6736(20)32553-8

[25] Hopkins DR Ruiz-Tiben E Weiss AJ Withers PC Eberhard ML Roy SL, 2013. Dracunculiasis eradication: and now, South Sudan. Am J Trop Med Hyg 89: 5–10. 23843492 10.4269/ajtmh.2013.13-0090 PMC3748487

[26] World Health Organization Collaborating Center for Dracunculiasis Eradication, 2020. Guinea WormWrap-up #272. Available at: https://www.cartercenter.org/resources/pdfs/news/health_publications/guinea_worm/wrap-up/272.pdf. Accessed February17, 2021.

[27] Thornton PK Kruska RL Henninger N Kristjanson PM Reid RS Atieno F Odero AN Ndegwa T, 2002. Mapping Poverty and Livestock in the Developing World. Washington, DC: IRLI.

[28] Food and Agriculture Organization of the United Nations, 2006. Policies and strategies to address the vulnerability of pastoralists in sub-Saharan Africa. Pro-Poor Livestock Policy Initiative. Nikola Rass, ed. *Chapter 3: Pastoralism in East and West Africa*. Rome, Italy: FAO, 7–13.

[29] Jean-Richard V Crump L Daugla DM Hattendorf J Schelling E Zinsstag J, 2014. The use of mobile phones for demographic surveillance of mobile pastoralists and their animals in Chad: proof of principle. Glob Health Action 7: 23209. 24499744 10.3402/gha.v7.23209 PMC3915884

[30] World Health Organization Collaborating Center for Dracunculiasis Eradication, 2020. *Guinea Worm Wrap-Up #274*. Available at: https://www.cartercenter.org/resources/pdfs/news/health_publications/guinea_worm/wrap-up/274.pdf. Accessed February 17, 2021.

[31] Durrant C 2020. Population genomic evidence that human and animal infections in Africa come from the same populations of *Dracunculus medinensis*. PLoS Negl Trop Dis 14: e0008623. 33253172 10.1371/journal.pntd.0008623 PMC7728184

[32] Boyce MR Carlin EP Schermerhorn J Standley CJ, 2020. A One Health approach for Guinea worm disease control: scope and opportunities. Trop Med Infect Dis 5: 159. 10.3390/tropicalmed5040159 PMC7709623 33066254

[33] Tayeh A Cairncross S Maude GH, 1993. Water sources and other determinants of dracunculiasis in the northern region of Ghana. J Helminthol 67: 213–225. 8288853 10.1017/s0022149x00013158

[34] Rubenstein BL 2021. Community-based Guinea worm surveillance in Chad: evaluating a system at the intersection of human and animal disease. PLoS Negl Trop Dis 15: e0009285. 33735242 10.1371/journal.pntd.0009285 PMC8023463

