# Supplementary material for: Surveillance of Human Guinea Worm in Chad, 2010–2018
Source: Am J Trop Med Hyg. 2021 May 24;105(1):188–95. doi: 10.4269/ajtmh.20-1525 (PMC8274751; doi:10.4269/ajtmh.20-1525)
Supplement: Supplementary file 1 [file tpmd201525.SD1.pdf]

Supplemental Table S1. Summary of variables available by surveillance year.

Variable	Years available	Effective Observations	Missing Observations	Total
**Worm-level variables**				
Worm containment (yes/no)	2012–2018	137	37	174 worms
Date of worm emergence date*	2010–2018	174	0	174 worms
Date of worm extraction	2012-2018	136	38	174 worms
Date of worm detection	2012-2018	137	37	174 worms
Location of worm emergence on patient's body	2012–2018	137	37	174 worms
Late detection of lesion	2014-2018	107	67	174 worms
Late or irregular bandaging	2014-2018	107	67	174 worms
Case possibly contaminated water	2011–2012, 2014–2018	134	40	174 worms
Worm not initially confirmed by a supervisor	2014-2018	107	67	174 worms
**Case-level variables**				
Age	2010–2018	111	0	111 cases
Sex	2010–2018	111	0	111 cases
Ethnicity	2012–2018	91	20	111 cases
Worms per person	2012–2018	111	0	111 cases
Case containment (yes/no)	2010–2018	111	0	111 cases
Occupation	2010–2018	111	0	111 cases
Case-patient travel history‡	2010–2018	111	0	111 cases
Case-patient entered water	2010–2011, 2014–2018	83	28	111 cases
Date admitted to health center	2012–2018	91	20	111 cases
Date discharged from health center	2012–2018	89	22	111 cases
Village of detection	2010-2018	111	0	111 cases
Case imported from another Region (yes/no)	2010-2018	111	0	111 cases
**Village-level variables**				
GPS coordinates	2010-2018	79	10	89 villages
Potable water available in village	2013-2018	64	25	89 villages

*For the years 2010–2011, worm emergence date was only recorded for the first worm in individuals with multi-worm infections. The day of the month was not recorded.

‡Information on travel history was collected as an independent question during 2012–2018. For the years 2010–2011, we inferred travel history using information about the village of case detection, the village of residence, and the village of worm emergence

Supplemental Table S2. Proportion of cases contained by surveillance level at time of detection (active vs. passive), 2010-2018.

Surveillance Level	N	n	(%)	p*
Passive surveillance†	55	15	(36.6)	**0.0084**
Active surveillance†	48	26	(63.4)	

Bold indicates statistical significance ($p < 0.05$).
*Fisher's exact test p-value.
†Active surveillance is carried out in areas with Levels 1 or 2 surveillance. Passive surveillance is carried out in Level 3 areas.

Supplemental Figure 1

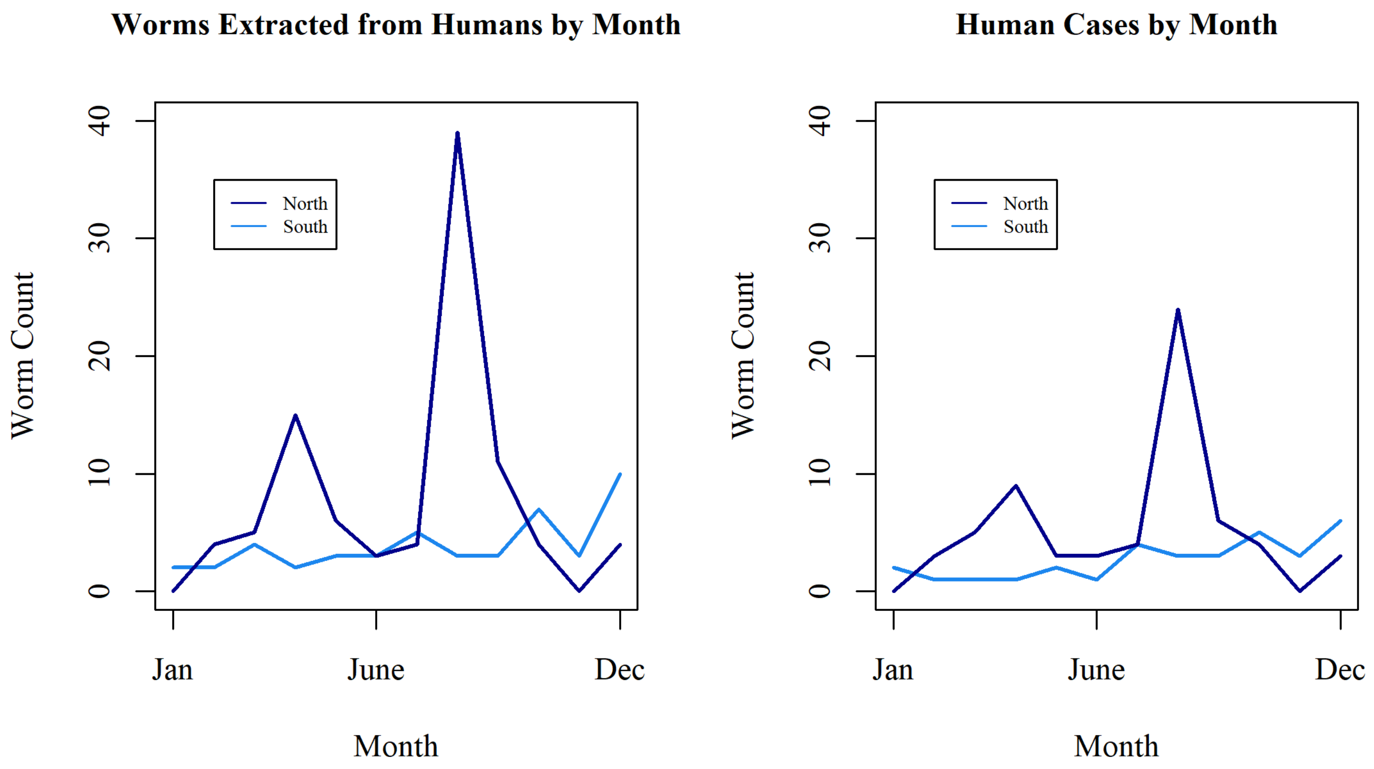

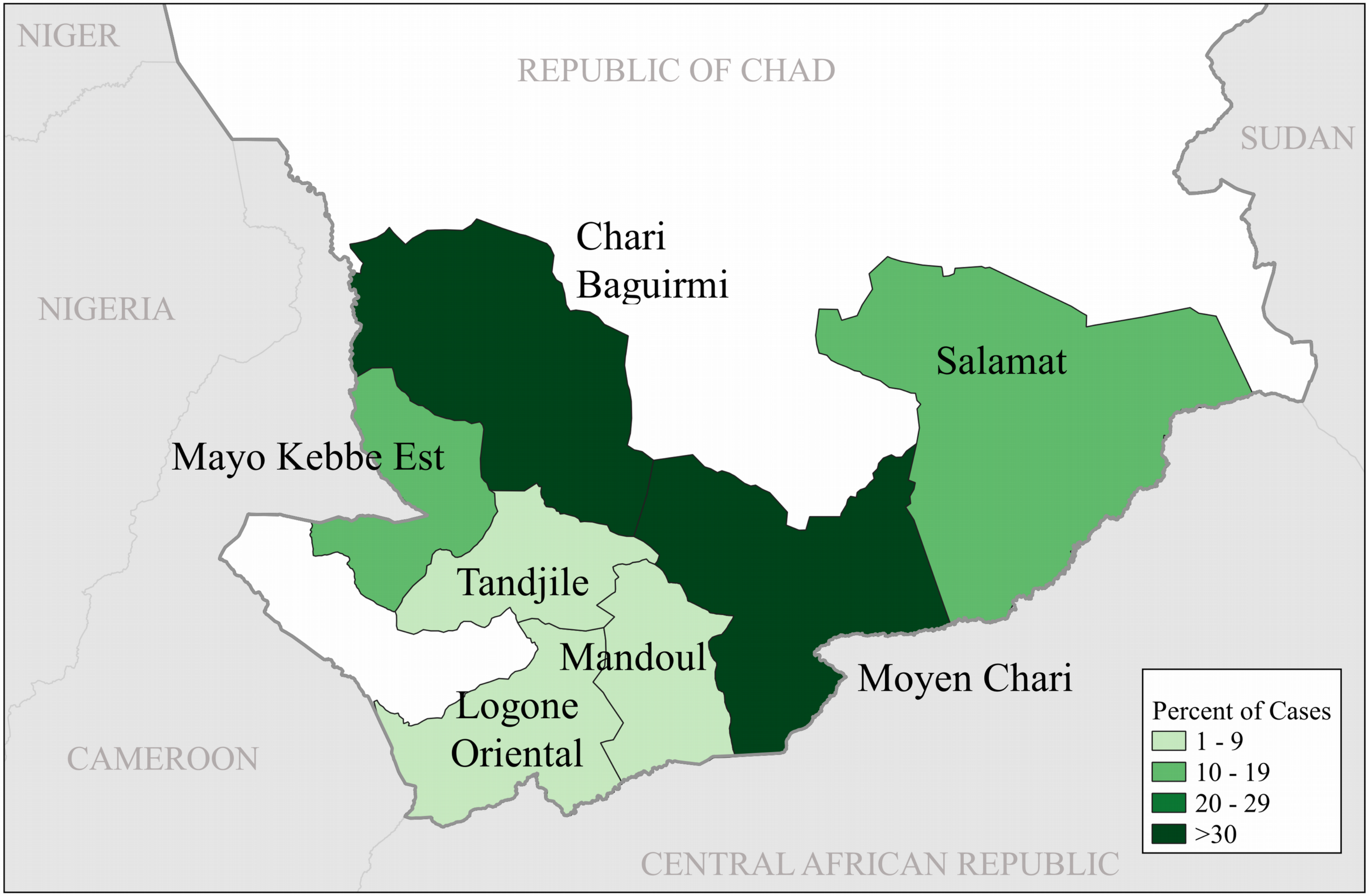

Supplemental Figure 2

Supplemental Figure S1. Numbers of Guinea worms and human cases detected by month in the northern and southern Chari River areas, Chad, 2010-2018. In the northern Chari River area, the peak numbers of worms and cases occurred in the months of April and August.

Supplemental Figure S2. Percent of human Guinea worm cases by Region within Chad, 2010-2018. Human cases were reported from seven different Regions within Chad, with most occurring in Moyen Chari and Chari Baguirmi Regions.
